# Effects of Multi-Deficiencies-Diet on Bone Parameters of Peripheral Bone in Ovariectomized Mature Rat

**DOI:** 10.1371/journal.pone.0071665

**Published:** 2013-08-16

**Authors:** Thaqif El Khassawna, Wolfgang Böcker, Parameswari Govindarajan, Nathalie Schliefke, Britta Hürter, Marian Kampschulte, Gudrun Schlewitz, Volker Alt, Katrin Susanne Lips, Miriam Faulenbach, Henriette Möllmann, Daniel Zahner, Lutz Dürselen, Anita Ignatius, Natali Bauer, Sabine Wenisch, Alexander Claus Langheinrich, Reinhard Schnettler, Christian Heiss

**Affiliations:** 1 Laboratory of Experimental Trauma Surgery, Justus-Liebig University, Giessen, Germany; 2 Department of Trauma Surgery, University Hospital of Giessen-Marburg, Giessen, Germany; 3 Department of Radiology, University Hospital of Giessen-Marburg, Giessen, Germany; 4 Animal Laboratory, Justus-Liebig University of Giessen, Giessen, Germany; 5 Institute of Orthopedic Research and Biomechanics, Centre of Musculoskeletal Research, University of Ulm, Ulm, Germany; 6 Department of Diagnostic and Interventional Radiology, BG Trauma Hospital Frankfurt/Main, Frankfurt, Germany; 7 Department of Veterinary Clinical Sciences, Clinical Pathology and Clinical Pathophysiology, Justus-Liebig University Giessen, Giessen, Germany; 8 Department of Veterinary Anatomy, Justus-Liebig University of Giessen, Giessen, Germany; Institut de Génomique Fonctionnelle de Lyon, France

## Abstract

Many postmenopausal women have vitamin D and calcium deficiency. Therefore, vitamin D and calcium supplementation is recommended for all patients with osteopenia and osteoporosis. We used an experimental rat model to test the hypothesis that induction of osteoporosis is more efficiently achieved in peripheral bone through combining ovariectomy with a unique multi-deficiencies diet (vitamin D depletion and deficient calcium, vitamin K and phosphorus). 14-week-old Sprague-Dawley rats served as controls to examine the initial bone status. 11 rats were bilaterally ovariectomized (OVX) and fed with multi-deficiencies diet. Three months later the treated group and the Sham group (n = 8) were euthanized. Bone biomechanical competence of the diaphyseal bone was examined on both, tibia and femur. Image analysis was performed on tibia via µCT, and on femur via histological analysis. Lower torsional stiffness indicated inferior mechanical competence of the tibia in 3 month OVX+Diet. Proximal metaphyseal region of the tibia showed a diminished bone tissue portion to total tissue in the µCT despite the increased total area as evaluated in both µCT and histology. Cortical bone showed higher porosity and smaller cross sectional thickness of the tibial diaphysis in the OVX+Diet rats. A lower ALP positive area and elevated serum level of RANKL exhibited the unbalanced cellular interaction in bone remodeling in the OVX+Diet rat after 3 month of treatment. Interestingly, more adipose tissue area in bone marrow indicated an effect of bone loss similar to that observed in osteoporotic patients. Nonetheless, the presence of osteoid and elevated serum level of PTH, BGP and Opn suggest the development of osteomalacia rather than an osteoporosis. As the treatment and fracture management of both osteoporotic and osteomalacia patients are clinically overlapping, this study provides a preclinical animal model to be utilized in local supplementation of minerals, drugs and growth factors in future fracture healing studies.

## Introduction

Osteoporosis is a common health problem characterized by low bone mass and structural deterioration of bone resulting in an increased susceptibility to fractures [1].

Bone matrix formations as well as general bone health are largely affected by mechanical loading and nutrition. Most important of these are calcium and vitamin D. Disorder of calcium balance could lead to Hypercalciuria which is often accompanied bone demineralization and vitamin D deficiency [2]. Beside its critical role in bone metabolism, vitamin D insufficiency was reported to add a significant additional fracture risk for osteoporosis. The status of vitamin D is crucial to allow the active absorption of calcium in the gut [3]. Therefore, limited or permitted nutritive diets can ultimately determine bone mass. Another essential element is phosphorus which is required for mineralization during bone formation. Vitamin K role in bone metabolism and protection against osteoporosis was also reported [4]. Furthermore, among the elderly osteomalacia might be associated with osteoporosis [5]. Osteomalacia is typically caused by lack of vitamin D leading to deficient mineralization of osteoid [6].

Several rat models of osteoporosis are described in the literature. Aged rats were utilized as models of senile osteoporosis. Nonetheless, trabecular bone volume of the vertebra of rats reportedly does not exhibit a decrease at 12 months of age [7]–[10]. Caloric restriction (CR) models were also described with debatable results, accumulated data indicated that CR delays the progression of age related disorders and could, therefore, impact age-related bone loss [11]. Other studies showed that lifelong CR led to bone loss via suppression of elevated parathyroid hormone (PTH) serum level [12]. Moreover, CR starting at 17 months of age caused femoral bone loss in Lobund Wistar rats [13]. These studies indicate that the CR effect is dependent on strain and experimental setting.

The ovariectomy approach to generate postmenopausal osteoporotic animal models was applied in rodents and mainly in rats [14]–[18]. Many studies focus on the sole effect of ovariectomy on the osteoporosis [15], [16], [19]–[22]. Nonetheless, those studies investigated the sole effect of ovarian hormone deficiency and neglected the multi-factorial nature of bone loss, especially the dietary factors. Other studies showed no markedly effect of combining Vitamin D deficiency with ovariectomy [23], also ovariectomy and low calcium diet did not cause a significant bone loss [24]. This rat model of postmenopausal osteoporosis is the first study investigating the combined effects of ovariectomy with a diet depleted of vitamin D and deficient of vitamin K, calcium, phosphorus. Most described models of osteoporosis focus on the spine, despite that several studies reported higher osteoporotic fracture risk in metaphyseal regions of long bone such as the distal radius, proximal humerus, and proximal femur [25]–[27]. Recently the authors published a new metaphyseal fracture model [28], which focused on establishing a critical sized defect to serve in testing biomaterial.

Taken all these points into consideration, the purpose of the present study was to explore the combined influence of bilateral ovariectomy and multi-deficiencies diet on bone quality and geometry in young adult rats as a reproducible and valid model of osteoporosis. Moreover, the study aims to rectify weather such an influence could lead to an osteomalatic phenotype. Nonetheless, the advantage of such a model would be the possibility to investigate biomaterial-aided fracture healing especially in the metaphyseal bone.

## Materials and Methods

### Experimental Design

This study is a part of trans-regional project concerned with the establishment of clinically relevant osteoporotic rat model as the foundation for biomaterial- aided healing of metaphyseal osteoporosis fractures. Recently, we have reported the osteoporotic bone status of these particular animal populations used for this experimental setup through bone mineral density (BMD) and bone mineral content (BMC) measurement by means of dual-energy X-ray absorptiometry (DEXA) as well as the evaluation of T-score and Z-score [29].

32 female Sprague-Dawley rats of (200–250 g) and 14 weeks of age were randomly divided into 3 groups: (i) control group (Control, also 0 M, n = 10), this non treated group served as control to evaluate the initial condition of bone parameters and as the baseline young healthy animal in the T-Score calculation; (ii) Sham-operated group (Sham, 1 M, n = 3; and 3 M, n = 8); (iii) ovariectomized+multi-deficiencies diet (OVX+Diet, 1 M, n = 3; and 3 M, n = 8), which was supplemented with deficient diet.

This study was performed in full compliance with our Institutional and German protection laws and approved by the ethical commission of the local governmental institution (“Regierungspräsidium Giessen”, permit number: 89/2009).Animals received intravenous injection of 62.5 mg/kg B. wt. ketamine (Hostaket®, Hoechst) and 7.5 mg/kg B. wt. xylazine (Rompun®, Bayer) for anesthesia prior to the bilateral ovariectomy and the Sham operations. Pain killers were delivered before surgery and up to five days after surgery subcutaneously using Metacam® (Meloxicam, BoehringerIngelheimPharma GmbH, Ingelheim, Germany) with 0.2 mg/kg dosage. No further pain killer or antibiotics were given post operatively, body weight was measured. Bone length of whole skeleton, femora and tibia were also controlled on the radiographic pictures (Table 1).

**Table 1:** Weight and bone length of different groups at all time points.

Group		N	body weight (g)	Femora length (mm)	Tibia length (mm)	Skeleton length (mm)
Control	0 M	10	363.8±61.7	142.3±5.72	176.41±15.1	882.17±34.7
Sham	3 M	8	361.1±70.1	150.1±8.26	188.86±11.9	889.89±22.6
OVX+Diet	3 M	8	331.3±47.2	146.15±5.35	195.85±10.6	888.36±23.3

**Figure 1:**
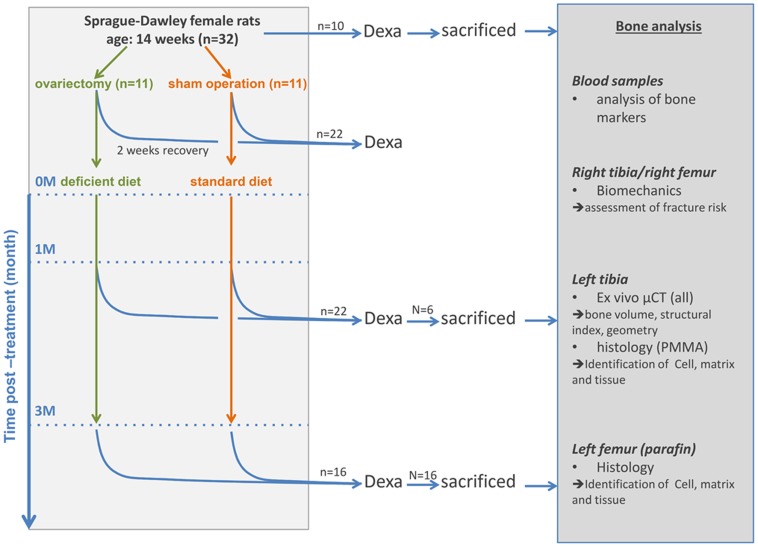
A chart depicting the work plan and experimental design. Female Sprague-Dawley rats were utilized for this experiment, 10 of which were sacrificed, analysis on these animals were carried out to obtain an initial bone status. Eleven animals were then ovariectomized (OVX), and another eleven rats were Sham operated (Sham) and animals were left to recover for two weeks. After recovery OVX animals were given a multi-deficiencies-diet where Sham animals received a standard diet. One month (1 M) and three months (3 M) after dietary treatment begun, 3 and 8 animals were sacrificed, respectively. Rats were scanned by DEXA at every time point and DEXA results were reported elsewhere. Besides DEXA, at 1 M only µCT analysis was performed on the left tibia. At both 0 M and 3 M Left tibia was analyzed in µCT before performing undecalcified histology; right femur and right tibia were tested biomechanically, and right femur was used for decalcified histology.

This study is focused on long bones; therefore, different samples from the same animal were utilized for different analysis. Left tibia was scanned in µCT before performing undecalcified histology; right femur and right tibia were tested biomechanically, and right femur was decalcified for paraffin analysis as histomorphometry and enzymo-histochemistry. An overview of the workflow is provided in Fig. 1.

### Animal Diet

The experimental group OVX+Diet rats, received a diet deficient in several elements when compared to the standard diet (Altromin-C1034 and C100, respectively; Altromin-Spezialfutter GmbH, Germany, Table S1). In a ratio to the standard diet received by the control group, the diet contained 0% vitamin D, 15% Calcium, 7% phosphorus, 50% vitamin K and 75% potassium. However, there are no caloric differences between the deficient diet and the normal diet.

### Assessment of Bone Quality using Biomechanical Testing

Biomechanical competence was performed to evaluate bone quality of the cortical bone in the tibia and femur. Right tibia and right femur (0 M and 3 M, n = 8/group for both) were harvested then incubated in sodium chloride saline and stored at −20°C. Prior testing, the bones were thawed and all tests run at 20°C room temperature. The specimens were kept moist by saline solution throughout the biomechanical experiments. Torsional testing was then performed, which mainly addresses cortical bone that would be affected in a longer term study and has its limitation to assess trabecular bone targeted in this study. Nonetheless it is known that trabecular bone significantly contributes to mechanical properties of bone under torsional loads a corresponding test on the tibiae was conducted to assess the torsional stiffness and failure torque. Briefly, both ends of the tibial bones were embedded in Polymethyl Methacrylate (PMMA) (Technovit 3040, HeraeusKulzer, Darmstadt, Germany). The bones were tested in internal rotation using a torsion apparatus in a materials testing machine (Z10, Zwick, Ulm, Germany). A vertical displacement was applied to a lever arm (50 mm) at a quasi-static displacement rate of 20 mm/min. The required force to displace the lever arm was registered by a 50 N load cell (KAP-Z, A.S.T., Germany). The torsion angle was calculated by the vertical displacement and the length of the lever arm. After a pre-load cycle, the test was repeated three times for each bone up to a maximum vertical force of 1 N and the torsional stiffness (Nmm/°) was calculated from the last loading cycle between 5 and 40 Nmm torsional moment. Finally, the bones were tested to failure and the breaking moment (Nmm) determined.

Additionally a bending test on the femurs was done to evaluate whether the cortical bone was already affected, which is known to happen at a later stage of osteoporosis [3].The femora were tested by a 3-point bending experiment assessing the bending stiffness and breaking load of the femora as described earlier [19]. The distal end of the femoral bones was also embedded in PMMA. Subsequently the femora were placed in customized 3-point bending rig at a support width of 32 mm. A central bending load was applied at a displacement rate of 1 mm/min. After two pre-load cycles up to 5 N a third cycle up to 20 N was carried out. The bending stiffness (N/mm) was determined from the third cycle. Finally a load to failure test revealed the breaking load (N) of the femora.

### Quantitative µCT Evaluation of Bone Parameters

To assess bone geometry, structural index and volume left tibia (controls at 0 M, n = 8; Sham and OVX+Diet at 1 M, n = 3 each; and Sham and OVX+Diet at 3 M, n = 8 each) were analyzed using micro-computed tomography (µCT). Bones were stored in parafilm to prevent dehydration during the scan. Samples were scanned in a µCT device manufactured and developed by SkyScan (SkyScan1072_80kV, Kontich, Belgium). The X-ray system is based on a micro focus tube (20–80 kV, 0–100 µA) reaching a minimum spot size of 8 µm at 8 W generating X-rays in cone-beam geometry. Similar systems have been described in detail before. Relative position of the object to the source determines geometric magnification and thus the pixel size. For our system, the manufacturer gives a maximum spatial resolution of 8 µm at 10% modulation transfer function (MTF), which is an accepted method for definition of the experimentally determined resolution and performance of an optical system. The X-ray detector consists of a 12-bit digital CCD high-resolution (1024×1024 pixel) camera with fiber optic 3.7∶1 coupling to an X-ray scintillator and digital frame-grabber.

In our experimental setting samples were positioned on a computer controlled rotation stage and scanned 180° around the vertical axis in rotation steps of 0.45 degrees at 75 kV. Raw data were reconstructed with a modified Feldkamp cone-beam reconstruction modus resulting in two dimensional cross sectional images with an 8-bit gray-scale resolution.

Tibiae were scanned en-bloc with an isotropic spatial resolution of (9 µm)^3^ voxel size. For standardized morphometry, the metaphyseal volume of interest (VOI) was set distal to the epiphyseal cartilage with an offset of 0.5 mm and a longitudinal extension of 3 mm. The cortical bone was excluded by means of marking the outer bonds by free hand drawn regions of interest (ROI). The grey-scale threshold was set to 75 to separate bone from the surrounding tissue using an adaptive thresholding method. The following parameters as defined by Parfitt et al. (1987) were measured: relative bone volume to total volume (BV/TV), mean trabecular thickness (Tb.Th), mean trabecular separation (Tb.Sp), trabecular number (Tb.N), and structure model index (SMI). Thresholding, definition of ROI and VOI as well as semi-automated morphometric measurement were performed with the ScyScan-CT-analyzer software (CT An, SkyScan).

Region of interest (ROI) was defined as diaphyseal cortical with a distance of 9 mm distal to the growth plate of the tibial plateau. ROI thickness was 2 mm at z-direction. Thresholding was done twice for either quantification of cortical thickness or evaluation of porosity. Measurement of cortical thickness using centre line algorithms suffers from pores within bone leading to artificial thinning of the cortical shell. Therefore we used an algorithm sequence containing i) adaptive thresholding ii) pore closing followed by iii) measurement of thickness. For evaluation of porosity and eccentricity, adaptive thresholding was followed by quantification.

### Histological Analysis

Decalcified histological analyses were conducted as previously described [30], [31] on the left femora. Briefly, femora were harvested, freed from soft tissue and fixed in 4% PFA and then decalcified (4% PFA and 14% EDTA at 4°C for 4 weeks). After embedding, paraffin blocks were sectioned in 6 µm thick slices. Histomorphometry was performed on Hematoxylin and Eosin (HE) stained sections, at 0 M (n = 6), and 3 M both Sham (n = 5) and OVX+Diet (n = 6). 15 microscopic fields were randomly analyzed at the metaphysical region as previously described [32]. Measurement included trabecular bone and bone marrow fat, each was contoured individually with in the defined region of interest (ROI). Osteoblasts and osteoclasts activities were investigated with an Alkaline phosphatase (ALP) and Tartrate-resistant acid phosphatase (TRAP) staining, at 0 M and 3 M, n = 3/group. Briefly, sections were deparaffinized, for TRAP treated with Sodium Acetate buffer and incubated in Napthol-AS-TR phosphate (N6125-1G, Sigma, Germany) and Sodium Tartrate (Merck, Germany) at 37°C for 60 minutes, for ALP sections samples were treated with Tris and then incubated in BCIP/NBT phosphate substrate at 37°C for 60 minutes. Both were then visualized with NovaRED (Vector, SK4800, CA) and finally counter stained with Hematoxylin. Osteoclasts (TRAP-positive multinuclear cells located on the bone surface) and osteoblasts (ALP positive cells on bone surface) were counted in randomly selected (15–20 microscopic fields per section) with in the metaphyseal region.

Undecalcified tibia (n = 4) were processed after the µCT scanning, embedded in PMMA according to standard protocols [33]. Grindings were stained with Van Gieson/Von Kossa to differentiate unmineralized tissue from mineralized bone and a ratio of osteoid surface to bone surface (OS/BS) was measured and expressed in mean and standard deviation as previously described [34].

Image capturing used Axioplan 2 Imaging system (Carl Zeiss, Germany) associated to a DC500 microscope (Leica, Germany). Image evaluation was performed using program Image-Pro®-Plus (Weiss Imaging and Solutions GmbH, Germany). Region of interest encompassed the trabecular bone and cortical bone in the metaphyseal region, and bone marrow to define adipose tissue content; growth plates were excluded.

### Serological Analysis of Bone Markers

Serum markers were analyzed to monitor the treatment effect on bone physiology. Venus blood was collected and transferred to 2.0 cc plain tubes (Microtube 2 ml with cap, Lot 2080601, Sarstedt, Nümbrecht, Germany). Serum was prepared by centrifugation at 9000 *g* (Micro 120 centrifuge, Hettich GmbH, Tuttlingen, Germany) for 1 minute and stored in aliquots à 100 µl at −80°C until analysis.

Leptin, and RANKL serum concentrations were assayed in duplicates at the Immulite 1000 Immunoassay analyzer using a commercially available test (Rat bone panel 2, catalogue number MXRABN20N02005, Millipore GmbH, Schwalbach, Germany). Bone Gla protein (BGP), phosphoprotein1 (Opn), and Parathyroid hormone (PTH) concentrations were also measured using a commercial assay (Rat bone panel 3, catalogue number MXRABN300N02002, Millipore GmbH, Schwalbach, Germany). TRAP5b serum concentrations were measured using a commercially available species specific enzyme immune assay (Rat TRAP-5b, Nittobo, Shiojima, Japan) according to the manufactureŕs instruction using an ELISA reader (PR 31000 TSC, Biorad, Munich, Germany).

### Statistical Analysis

Results were presented as mean ± standard error of the mean (SEM). All data were analyzed using the Statistical Package PASW 20.0 (SPSS Inc., USA) using Mann-Whitney U test for unpaired nonparametric data with bonferroni correction for biomechanical testing and histomorphometry as well as the serum bone turnover markers. ANOVA followed by bonferroni correction test were used for the µCT data. The Kolmogorov-Smirnov test was used to test normality. P-values of less than 0.05 were chosen to indicate significance.

## Results

### Multi-deficiencies Diet Affects Functional Competence of the Tibia

**Figure 2:**
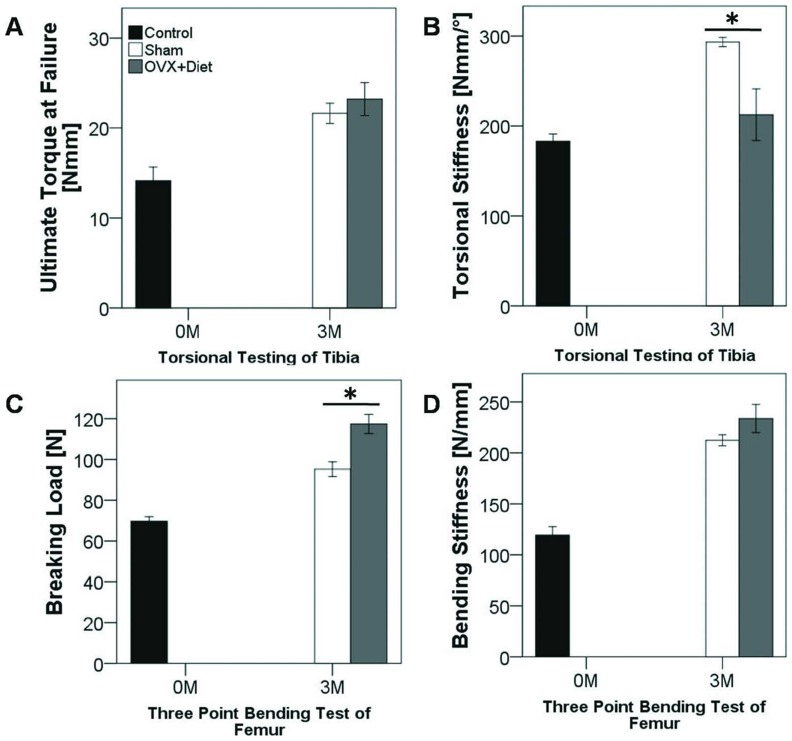
Biomechanical testing of tibia and femur of the ovariectomized rat after three months of multi-deficient diet treatment. (A) Ultimate torque at failure shows no significance between the groups at 3 M. (B) Tibia of treated rats showed lower torsional stiffness compared with the Sham operated rats. (C) Breaking load of the femur shows an increased needed load to break the femur of treated animals at 3 M. (D) Bending stiffness of the femur showed no significant difference between the treatment group and the Sham group. (* = p≤0.05, Mann Whitney U with bonferroni correction, n = 8 per group).

Osteoporosis is characterized by a decrease of bone tolerance to stress. Osteoporotic animal models shall reflect this criterion. Therefore, mechanical properties were examined to evaluate the function integrity of both tibial and femoral bones. Despite no statically significant difference was found in the ultimate torque at failure (Fig. 2A), tibia showed a lower torsional stiffness when compared to the Sham animals after 3 M of treatment (Fig. 2B). On the other hand breaking load of the femur (Fig. 2C) increased in the OVX+Diet at 3 M, whilst bending stiffness showed no significant difference in femora between the two groups (Fig. 1D). Interestingly, at 3 M both groups show increased biomechanical competence when compared to the initial status at 0 M, indicating that bone growth continuum has not reached bone peak mass at time point 0 M.

### Deprivation of Tibia Trabeculae in the OVX+Diet Group After Three Months of Treatment

**Figure 3:**
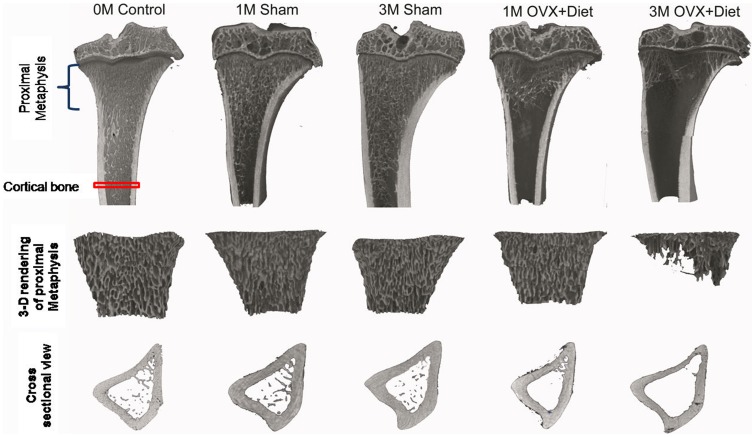
High resolution µCT defined tibia trabeculae as the impaired structural parameter. (Left panel), 3D reconstruction images reflect the progressive effect of combined ovariectomy and deficient diet treatment. (Top right panel) at 1 M OVX+Diet showed less trabeculae in the metaphyseal region than 1 M Sham and 0 M control groups; at 3 M trabecular loss becomes more severe in the OVX+Diet when compared with 3 M Sham group, thus indicating trabecular bone susceptibility to the treatment. (Bottom right panel) Representative 3D images show affected cortical bone (thinner) and porosity in cross sections of rat tibia in OVX+Diet at 3 M.

In order to visually and quantitatively determine how peripheral bone structural components are affected at the metaphyseal region by a multi-deficient diet and the estrogen depletion due to the ovariectomy, tibias were analyzed at two consecutive stages post induction by means of µCT (Fig. 3 top).

The trabecular 3D rendering (Fig. 3, middle), depicts the differences in the metaphyseal region between time points and groups. At the initial time point, trabecular bone appeared compact and normally distributed in the metaphyseal region and in the proximal parts of the diaphysis (Fig. 3, 0 M Control). Further, at 1 M time point trabecular bone in the Sham group was also well distributed in the metaphyseal region and also reached the proximal parts of the diaphysis (Fig. 3, 1 M Sham), the trabecular formation appears even to increase in the Sham group from 1 M to 3 M. However, the effect of the treatment was visible at 1 M OVX+Diet, trabecular structure filled only the metaphyseal region and was absent in the proximal diaphyseal part. This clear loss of trabeculae was more prevalent at 3 M in the OVX+Diet; trabecular bone appeared disintegrated when compared to the 1 M OVX+Diet and the 3 M Sham. The longitudinal 3D reconstruction shows also an intriguing effect of the treatment in the epiphyseal region were it appears in the OVX+Diet at 3 M less compact and even smaller in size. The treatment effect was also visible, although in less severity, on the cortical bone especially at 3 M OVX+Diet (Fig. 3, bottom).

**Figure 4:**
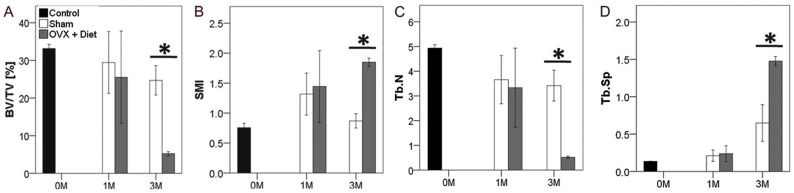
Qualitative analysis of trabecular bone shows inferior bone quality resulting from multi-deficiencies diet combined with bilateral ovariectomy in rats after 3 **M.** (A) BV/TV showed how bone tissue is affected within the total volume; these results suggest that at 3 M the treatment results in less mineralized tissue in OVX+Diet when compared to the 3 M Sham. (B) Structure Model Index (SMI) indicated the trabecular shape change at 3 M in the OVX+Diet group. (C) Tb. N was lower in the 3 M OVX+Diet group compared to the Sham group. (D) Higher trabecular separation in the OVX+Diet group when compared to the Sham after 3 M of treatment. (* = p≤0.05, one-way ANOVA with bonferroni correction, 0 M, n = 8; 1 M, n = 3 per group; 3 M n = 8 per group).

However, qualitatively, the ratio of bone volume over the total volume implicates the percentage of bone tissue within the whole scanned volume. At the initial status 0 M control group BV/TV [%] was highest. Despite the lower values of Sham and OVX+Diet at 1 M, no significant difference was seen in comparison to the 0 M control group. Intriguingly at 3 M, the OVX+Diet drastically declined to less than 10% BV/TV, and the Sham group showed minimal decrease with no statistical significance (Fig. 4A). However, the change in the trabecular shape could be interpreted through the Structural Model index (SMI). The closer to zero the model indicate plate-like trabeculae, moving toward three the index implies a rod-like trabecular shape. At 0 M control the initial bone status showed an SMI around 0.75, the SMI increased to about 1.4 at 1 M in Sham and OVX+Diet. Moreover, the SMI at 3 M increased significantly in the OVX+Diet when compared with either the 3 M Sham or the 0 M control (p≤0.05, for both). Interestingly, Sham group decreased at 3 M to a value close to the 0 M control (Fig. 4B). Moreover, trabecular number (Tb.N), trabecular separation (Tb.Sp), which are decisive parameters of trabecular bone quality, were bother significantly lower in the 3 M OVX+Diet rats when compared to either the 0 M control group or the 3 M Sham rats (p≤0.005, for all). Other parameters as TV, BV and trabecular thickness also show significant impairment in the 3 M OVX+Diet (Fig. S1).

### The Effect of OVX+Diet on Cortical Bone Structural Properties

**Figure 5:**
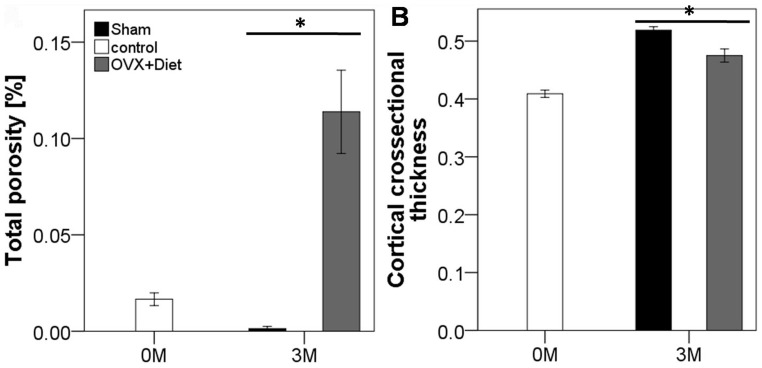
µCT analysis of cortical bone parameters show affected porosity and thickness in the OVX+Diet treatment after 3 months. (A) Porosity measurement showed that both groups; the 0 M control and the 3 M Sham have significantly lower porosity than the 3 MOVX+Diet. (B) Cortical thickness was significantly higher in the 3 M Sham group when compared to either 3 M OVX+Diet or 0 M control group. (* = p≤0.05, Mann Whitney U with bonferroni correction, n = 8 per group).

Cortical bone properties, as analyzed by µCT are exhibited in Fig. 5. The 3 M OVX+Diet demonstrated significantly higher tibial total porosity than both the 3 M Sham group and the 0 M control group. The 3 M Sham group exhibited also significantly lower porosity than the 0 M control group. Cortical cross-sectional thickness was also higher at 3 M in the Sham group than the OVX+Diet and also than 0 M control. However, the OVX+Diet group showed at 3 M higher cortical thickness than the 0 M control group.

### Larger Trabecular Area and Adipose Tissue Accumulation After 3 M of Treatment

**Figure 6:**
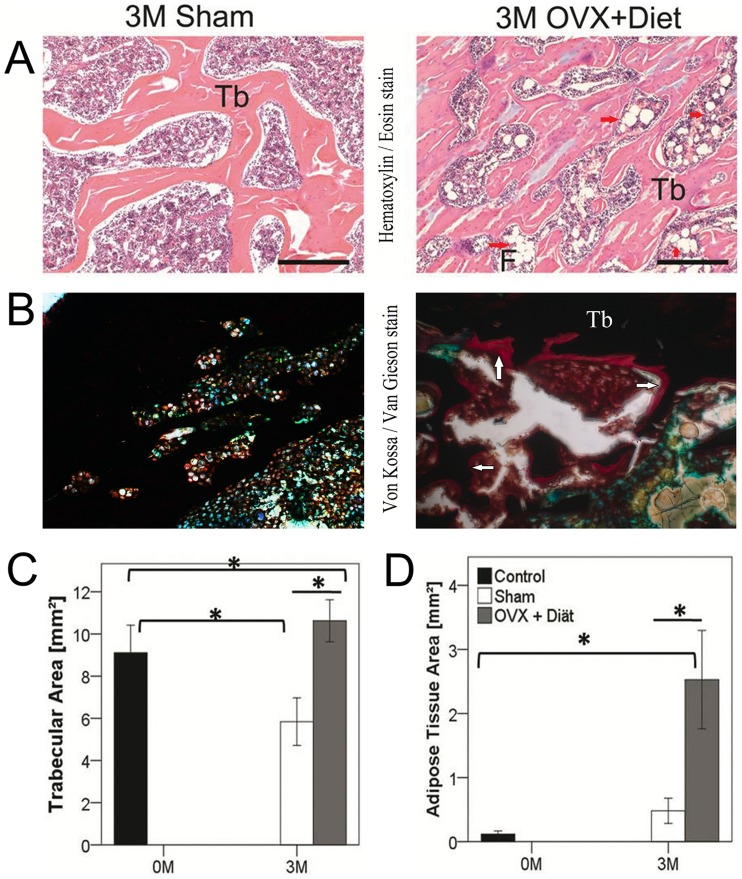
OVX+Diet group has increased trabecular and adipose tissue area, and no unmineralized osteoid area. (A) HE staining of proximal femur metaphyeal region of Sham operated animals (left) showed a comparable trabecular area to the 0 M control, no adipocytes were identified. OVX+Diet (right) showed larger areas covered with trabeculae and abundant adipocytes around the trabecula. (B) Von Kossa/Van Gieson staining of Sham operated animals (left) showed no unmineralized bone matrix (osteoid) on the surface the trabecular bone, OVX+Diet (right) showed osteoid at the surface of the trabeculae in the proximal metaphyseal region of the tibia. (C) Quantitative histomorphometry showed an increase in trabecular area in the 3 M OVX+Diet group, the 3 M Sham trabecular area decreased in comparison to the 0 M control. (D) Area of adipose tissue increased in the 3 M OVX+Diet when compared to 0 M control group and the 3 M Sham group, nonetheless, no change in the 3 M Sham. (* = p≤0.05, Mann Whitney U with bonferroni correction, n = 8 per group, scale bar = 50 µm, Tb = trabecular bone, F = Adipose tissue, red arrows fat tissue, white arrows osteoid).

Hematoxylin and Eosin (HE) stain was used to qualitatively examine morphological changes in the trabecular structure of the tibia. Histomorphometry was used to quantify trabecular number and trabecular area and adipose tissue. Descriptively, at 3 M OVX+Diet group showed more trabecular area than 3 M Sham. The HE stain also shows larger adipose tissue area around the trabecula (Fig. 6A). Histomorphometrical results confirmed the descriptive observations. Trabecular area was larger in the 0 M control when compared to 3 M in the Sham group, which also showed smaller trabecular area when compared to the OVX+Diet at 3 M (Fig. 6C). The adipose tissue area showed no change between the 0 M control and the 3 M Sham and but was larger at 3 M in the OVX+Diet group when compared with either 0 M control or 3 M Sham (Fig. 6D). However, no significant difference was seen in the trabecular number between the groups. Von Kossa/Van Gieson stain on undecalcified bone also showed a compact trabecular bone in the Sham group when compared with the OVX+Diet group at 3 M which showed unmineralized osteoid around the borders of the trabecular bone (Fig. 6B). Histomorphometry showed that almost 5 times more osteoid is covering the bone surface of the OVX+Diet group when compared to the Sham; OS/BS [group: mean± SD]; [Sham 0.7±0.02] versus [OVX+Diet 3.5±0.3].

### Lower Osteoblast Numbers in 3 M OVX+Diet Rats

**Figure 7:**
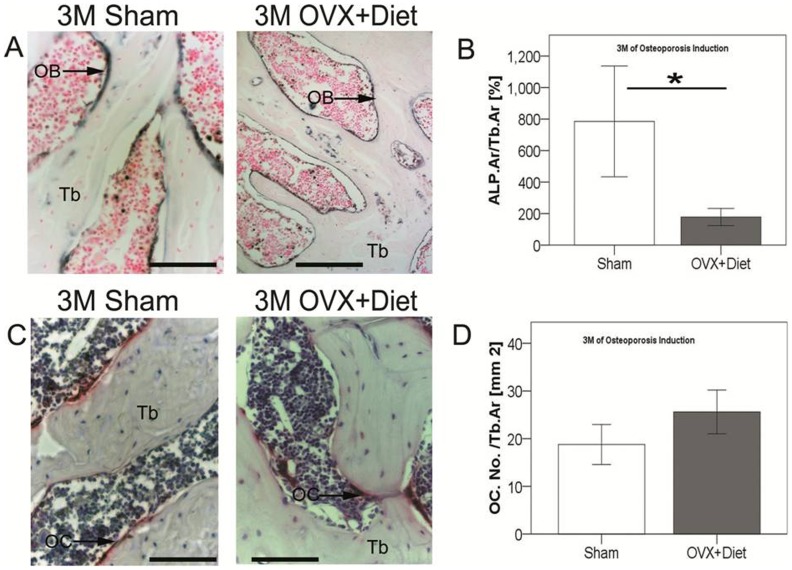
Unbalanced cellular populations contribute in 3 M OVX+Diet bone alteration. (A+B) 3 M OVX+Diet showed less activity of osteoblasts at the trabecular area when compared with 3 M Sham animals suggesting a lower bone formation. (C+D) Osteoclasts number at trabecular surface of OVX+Diet showed a higher trend than in the Sham group at 3 M indicating an increased bone resorption caused by the treatment. (* = (p≤0.05), Mann Whitney U with bonferroni correction, n = 3 per group 15 microscopic fields, scale bar = 5 mm, Tb = trabecular bone, OB = osteoblasts, OC = osteoclasts).

Histological analysis and µCT suggested loss of bone quality and deformation of trabecular bone in tibia of OVX+Diet rats. Therefore, bone sections were stained to define ALP activity, which mark osteoblasts. 3 M OVX+Diet showed a significantly lower osteoblast activity normalized to the trabecular area (Fig. 7A, B). On the other hand tartrate resistant acid phosphatase (TRAP) activity stain was performed to quantify osteoclast numbers and normalize them to the trabecular area. Despite TRAP positive cells were more abundant in the 3 M OVX+Diet when compared to the 3 M Sham, no statistical significance was seen (Fig. 7C, D; p = 0.054).

### Effect of OVX+Diet on Bone Turnover Markers in Serum

**Figure 8:**
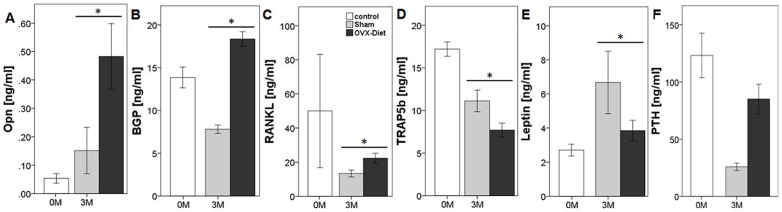
Biochemical markers reflect the bone metabolism. (A–D) Opn, BGP, RANKL, and PTH concentration in serum was higher in the 3 M OVX+Diet than the 3 M Sham. (D -F) lower concentrations of TRAP5b and leptin were seen in the OVX+Diet at 3 M compared to the Sham group. (* = (p≤0.05), Mann Whitney U with bonferroni correction, n = 8 per group).

Biochemical bone turnover markers reflect changes in bone metabolism. Bone markers taken into consideration in this study reflected bone anabolism and catabolism (Fig. 8). BGP show higher values at 3 M in the OVX+Diet than the Sham group which also shows lower concentrations than the 0 M control group. Serum Opn concentration was higher in the OVX+Diet group at 3 M when compared with 3 M Sham and 0 M control. Serum PTH levels was significantly increased in the OVX+Diet when compared with the sham after 3 M of treatment. Serum RANKL concentration was higher in the 3 M OVX+Diet group when compared to the 3 M Sham. However, lower serum TRAP5b concentration was seen in the 3 M OVX+Diet group than the 3 M Sham group. Moreover, Leptin was also lower in the OVX+Diet than the 3 M sham group.

## Discussion

Bone loss and decreased bone quality is the hallmark of osteoporotic bone. Recently we reported the lower BMD and BMC when examined by means of DEXA [29], which represent the golden standard to diagnose osteoporosis. In his study, further analysis to investigate the functional, structural and cellular changes in the peripheral bone was explored. Such investigation would provide the validity of employing a multi-deficiencies diet in combination to bilateral ovariectomy to establish a reproducible osteoporotic rat model for further use in fracture healing studies. Histological findings and µCT results both showed a higher trabeculae size in the OVX+Diet group; however, the histological analysis exhibited a higher adipocytes accumulation around the trabecula of the experimental group. The µCT parameters of the metaphyseal region also show the lower portion of bone tissue within the higher total tissue volume. Interestingly, the µCT analysis showed that the trabeculae lose begun after 1 M in of the treatment of OVX+Diet group. µCT analysis of the diaphyseal part of the bone reflected changes in the bone quality through higher porosity, lower and lower trabecular thickness in the 3 M OVX+Diet group compared to the Sham. These results supports the inferior functional competence resulted from the treatment as reflected by the biomechanical tests which showed lower torsional stiffness in the tibia of the 3 M OVX+Diet group when compared to the 3 M Sham.

The Food and Drug Administration (FDA) guidelines of animal models for osteoporosis beside many reviews meticulously investigated bone alteration of the mature rat at 3 M after OVX. [27], [35]. These reports have set the bone parameters of the OVX treatment of mature and aged rats. Moreover, based on the FDA guidelines, “The rat OVX model is useful for the evaluation of potential agents for the prevention of postmenopausal osteoporosis but not for agents for the treatment of osteoporosis”. The initial bone status (0 M control) indicates a healthy adult bone. Nonetheless, µCT parameters (BV/TV, SMI and Tb.N), showed higher values at 3 M in Sham animals when compared to the 0 M control group. This change indicates a continuing bone growth. On the other hand, lower values of the same parameters in the 3 M OVX+Diet group despite the continuing bone growth add to the treatment validity.

### Higher Trabecular Size and Volume but Less Bone and Mineralized Tissue in the Metaphyseal Region of 3 M OVX+Diet Rats

Imaging techniques showed, in the 3 M OVX+Diet group, higher size and volume of trabecular bone in both histological analysis (trabecular area, Fig. 6 C) and µCT (TV, Fig. S1A) when compared to the Sham group. The 3D high resolution µCT technique suggests and explanation for the higher trabecular volume and size through higher SMI value which is also indicative of osteoporotic effect as trabeculae changes from plat to rod like. Moreover, higher Tb.Sp, lower Tb.Th (Fig. S1 C) and lower Tb.N were reported to relate exponentially with TV and inversely with BV [36], [37]. These findings also accord with the reported results ovarian hormone deficiency in OVX rats, were decreased bone density and increased fragility due to decreased spongiosa bone volume were reported [20], [38]. Bone in OVX rats was also characterized by increased trabecular separation, reduced trabecular thickness, trabecular connectivity, and trabecular number [35].

Nonetheless, decalcified histographs and histomorphometry were recently reported to be misrepresentative of bone ratio in the trabecular area or the effects of Tb.Th. [39]. Therefore, Von Kossa/Van Gieson stain of undecalcified tissue was performed and showed unmineralized regions of trabecular bone only in the 3 M OVX+Diet group. This also explains the lower BV/TV ratio in the µCT analysis. Moreover, the higher levels of serum Opn indicate an increase of extracellular matrix protein production by immature and not mature osteoblasts [40]. Nevertheless, the presence of osteoid in the OVX+Diet rats and its absence in the Sham group, suggests the accumulation of non-mineralized bone matrix, which is a diagnostic indicative of osteomalacia rather than osteopenia or osteoporosis.

### Higher Adipose Tissue in the OVX+Diet Group Three Months After Treatment

Another intriguing observation in the histological analysis is bone marrow fat. This finding also in line with the previously reported DEXA results [29] concerning in body fat measurement *in vivo*, where a progressive increase in the fat% was described in the treatment group when compared with the Sham group.

There was no bone marrow fat in the 3 M Sham group but abundant adipocytes were located around the trabeculae of the OVX+Diet group at 3 M. Reports established the hematopoietic properties of bone marrow and the absence of bone marrow fat at any skeletal site in newborn infants. Nonetheless, adipocytes accumulate in bone marrow increases with age (up to 70% of marrow in individuals over 30 years of age. However, this age-related increase in marrow fat was associated with age-related bone loss and BMD, establishing the widely accepted view of an inverse correlation between these parameters [41]. This fact shows that in our model bone loss was likewise correlated to increment in the adipose tissue area. Moreover, estrogen plays a crucial role in the development of marrow adiposity and bone loss. Estrogen deficiency is reported to result in uncoupling of the bone remodeling units especially after the menopause, which is marked by an increase in marrow adiposity accompanied by a decline in bone mass [42], [43]. Estrogen also has a direct effect of estrogen on the mesenchymal stromal cells fate towards either osteoblasts or adipocytes [44], [45]. Moreover, limited diet could also contribute in the adipose tissue accumulation in bone marrow. Caloric restriction in young mice was reported to increase bone marrow adiposity and was associated with lower leptin serum levels [46]. In this study similar effect was noted as no significant difference in body weight was seen between the groups. However, lower serum levels of leptin together with the histomorphometrical analysis and DEXA results show higher bone marrow adiposity. These findings are suggested outcomes of vitamin D and calcium deficiency as vitamin D is reported to inhibit adipogenesis at the molecular level through a vitamin D receptor (VDR)-dependent inhibition [47].

### Unbalanced Bone Metabolism After 3 M of Dietary Treatment with Bilateral Ovariectomy in Rats

ALP staining showed a reduced ALP positive area in the OVX+Diet group at 3 M when compared to the Sham group. This measurement is however normalized on the total area of trabecular bone which is higher in the OVX+Diet than the Sham group. However, Serum Opn high levels indicate an affected mineralization process as Opn is a regulator of the mineralization process, expressed by osteoblasts in advance to mineral deposition [48]. On the other hand, the elevated serum levels of BGP indicate not only bone formation but BGP is also released from the extracellular matrix during resorption [49]. PTH is a key factor of bone remodeling that can lead to either anabolic or catabolic effects [50]. However, vitamin D serum levels relates inversely with PTH serum levels [51]. This suggests that immature osteoblasts were unable to mineralize the synthesized osteoid as seen in the Von Kossa/Van Gieson stain. Another explanation could be the impairment Vitamin K-depended carboxylation of BGP in blood, due to the deficient diet [52]. However, the elevated serum levels of both BGP and Opn were reported in patients with osteomalacia [53]. Collectively, these factors, with the elevated PTH serum levels under a vitamin D and calcium deficient diet indicate osteomalacia [50].

Interestingly, the TRAP staining did not show any significant difference between the 3 M Sham and 3 M OVX+Diet group. Nonetheless, elevated RANKL serum levels suggest the initiation of more resorption. In such a case, lower TRAP5b serum levels that should indicate bone resorption [49], could be explained as in the transient phase. In bone, vitamin D and PTH stimulate resorption indirectly by activation of osteoblasts synthesizing RANKL [49]. Depletion of vitamin D in the model described here may influence this activation, which may result in less osteoclast-recruitment for resorption.

### Inferior Diaphyseal Bone Quality of the OVX+Diet Rats at 3 M

The three-point bending test biomechanical testing is commonly used to evaluate intrinsic functional bone parameters. Bending stiffness thereby indicates the mechanical load of cortical bone [54]. This explains the insignificant discrepancy in the bending stiffness at 3 M. This goes in line with clinical studies which report that osteoporosis in humans affects trabecular bone earlier than cortical bone [55]. Furthermore, breaking load is related to the bone flexibility increased in the OVX+Diet animals suggesting a lower mineralization. On the other hand, the trabecular bone integrity influences the mechanical properties in a much higher degree under the torsion test [56]. Therefore, despite the increased bending stiffness OVX+Diet showed lower torsional stiffness when compared to the yet growing 3 M Sham. Moreover, lower bone quality indicated by higher porosity and lower cortical thickness in the current model in lines with the biomechanical competence test results. On the other hand, reduced serum leptin was reported to indicate reduced cortical bone thickness in a model of caloric restriction [57].

### Nutritional Aspects of Bone Health

Reports showed that OVX rats lack of estrogen suppression of RANKL production elevates its level in the serum [58] leading to the induction of osteoclasts progenitors. Nonetheless, through the increased bone resorption minerals, mainly calcium will be consequently lost from bone. Therefore, calcium supplementation was widely studied in OVX rodents and has proven to inhibit bone loss [59]–[61]. Furthermore, vitamins as vitamin D and vitamin K were reported to protect bone after ovariectomy by reducing the induced bone loss resulting from estrogen deprivation [62]–[66]. The model described here tried to reach severe bone loss by the either depletion of vitamin D and deficient calcium and vitamin K. The model also encourages the future investigation of age related osteometabolic diseases such as osteomalacia, which are not addressed in this study.

### Conclusion

Taken together, this novel treatment of multi-deficient diet combined with bilateral ovariectomy revealed the observation of severe bone loss after three months of the treatment. However, the presented results reflected diagnostic criteria of osteomalacia. Intriguingly, the model shows a predisposition of fat accumulation in the bone marrow which is reported for the first time in animal models osteomalacia lesions. Furthermore, diagnosis of both osteoporosis and osteomalacia in the clinic depends on the T-score and Z-score index obtained by DEXA and QCT. Therefore, an overlapping treatment and fracture management are mainly taken. This fact infers the validity of this model to be utilized to study fracture healing and the local delivery of minerals and growth factors on a bone with inferior via biomaterials.

## Supporting Information

Figure S1 (A) Total Volume (TV) increased at 3 M in Sham and OVX+Diet when compared with the initial status (0 M control group), however the treatment showed a greater affect on TV of OVX+Diet than the Sham at 3 M. (B) Bone volume defines bone tissue, compared with either the 0 M control or the 1 M Sham and OVX+Diet, 3 M OVX+Diet exhibited a drop in BV, whereas Sham group increased significantly at 3 M than 1 M. BV shows that the treatment has a direct effect on the bone tissue. (C) Trabecular thickness was affected within three months after treatment.
(TIF)

Table S1 Lists of main nutritional ingredients and their concentrations in both standard and multi – deficiencies diets.
(DOCX)
