# Supplementary figures and images for: Effects of Multi-Deficiencies-Diet on Bone Parameters of Peripheral Bone in Ovariectomized Mature Rat
Source: PLoS One. 2013 Aug 16;8(8):e71665. doi: 10.1371/journal.pone.0071665 (PMC3745426; doi:10.1371/journal.pone.0071665)

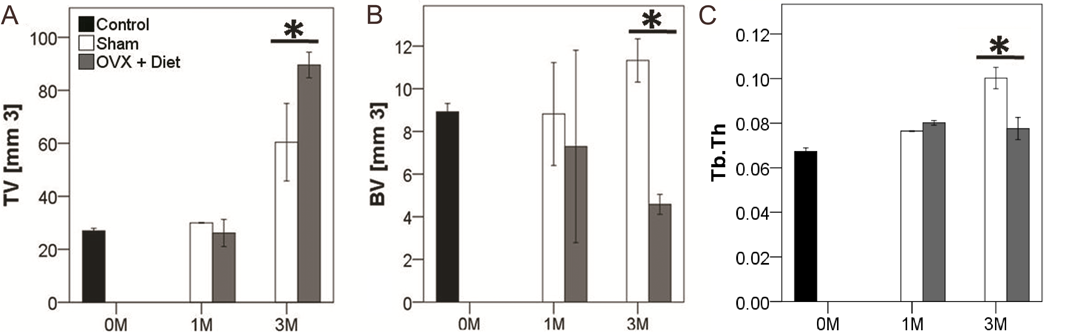

Supplement: Figure S1 — (A) Total Volume (TV) increased at 3 M in Sham and OVX+Diet when compared with the initial status (0 M control group), however the treatment showed a greater affect on TV of OVX+Diet than the Sham at 3 M. (B) Bone volume defines bone tissue, compared with either the 0 M control or the 1 M Sham and OVX+Diet, 3 M OVX+Diet exhibited a drop in BV, whereas Sham group increased significantly at 3 M than 1 M. BV shows that the treatment has a direct effect on the bone tissue. (C) Trabecular thickness was affected within three months after treatment. (TIF) [file pone.0071665.s001.tif]
