# Supplementary material for: Effects of Multi-Deficiencies-Diet on Bone Parameters of Peripheral Bone in Ovariectomized Mature Rat
Source: PLoS One. 2013 Aug 16;8(8):e71665. doi: 10.1371/journal.pone.0071665 (PMC3745426; doi:10.1371/journal.pone.0071665)
Supplement: Table S1 — Lists of main nutritional ingredients and their concentrations in both standard and multi – deficiencies diets. (DOCX) [file pone.0071665.s002.docx]

Table S 1: Ingredients of both standard and multi – deficiencies diet

| **Ingredients** | **Units** | **Content/normal** | **content / diet** |
| --- | --- | --- | --- |
| Alanine | mg/kg | 2528 | 8703.23 |
| aluminum | mg/kg | 3.706 | 3.566 |
| Arachidic C-20: 0 | mg/kg | 250 | 250 |
| Arachidonic acid C 20: 4 | mg/kg | 2.5 | 2.5 |
| Arginine | mg/kg | 9828.79 | 11513.058 |
| Aspartic acid | mg/kg | 3583.14 | 15052.732 |
| Behenic C-22: 0 | mg/kg | 250 | 250 |
| Benzoic acid | mg/kg | 100 | 100 |
| Biotin | mg/kg | 0.201 | 0.5 |
| Calcium | mg/kg | 9310.506 | 1367.728 |
| Capric acid C-10: 0 | mg/kg | 2.5 | 2.5 |
| Chlorine | mg/kg | 3630000 | 3943.798 |
| Choline chloride | mg/kg | 1011.5 | 1002.875 |
| Cobalt | mg/kg | 0.147 | 0.148 |
| Copper | mg/kg | 5.751 | 5.366 |
| Crude Ash | mg/kg | 54943.225 | 20165.35 |
| Crude Fat | mg/kg | 50830 | 50651 |
| Crude Fiber | mg/kg | 40450 | 40544.725 |
| Crude Protein | mg/kg | 176115 | 180682.875 |
| Cystine | mg/kg | 3196.18 | 4033.917 |
| Digest.Phosporus | mg/kg | 7199.565 | 475.212 |
| Disaccharide | mg/kg | 110960.5 | 98216.06 |
| Docosahexaenoic acid C22: 6 | mg/kg | 2.5 | 2.5 |
| Eicosadienoic C-20: 2 | mg/kg | 250 | 250 |
| Eicosaenoic acid C-20: 1 | mg/kg | 250 | 250 |
| Eicosapentaenoic acid C20: 5 | mg/kg | 2.5 | 2.5 |
| Energie/Metab. | kcal/kg | 3518.055 | 3662.779 |
| Erucic acid C-22: 1 | mg/kg | 2.5 | 2.5 |
| Fluorine | mg/kg | 4.17 | 3.584 |
| Folic acid | mg/kg | 10.0024 | 10.0006 |
| Glutamic acid | mg/kg | 23674.97 | 25377.87 |
| Glycine | mg/kg | 3136 | 5905.315 |
| Histidine | mg/kg | 5275.79 | 6190.55 |
| Inositol | mg/kg | 111 | 102.75 |
| Iodine | mg/kg | 0.514 | 0.396 |
| Iron | mg/kg | 178.579 | 179.188 |
| Isoleucine | mg/kg | 7222.82 | 8811.956 |
| Lauric acid C 12: 0 | mg/kg | 2.5 | 2.5 |
| Leucine | mg/kg | 14762.77 | 16897.134 |
| Linoleic C18: 2 | mg/kg | 35050 | 35050 |
| Linolenic C18: 3 | mg/kg | 150 | 150 |
| Lysine | mg/kg | 17400.97 | 15933.41 |
| Magnesium | mg/kg | 683.506 | 666.622 |
| Manganese | mg/kg | 100.888 | 99.984 |
| margaric | mg/kg | 2.5 | 2.5 |
| Methionine | mg/kg | 10688 | 7689.508 |
| Moisture | mg/kg | 81735.625 | 81323.725 |
| Molybdenum | mg/kg | 0.198 | 19.825 |
| Myristinsäue C-14: 0 | mg/kg | 2.5 | 2.5 |
| Nervonic C-24: 1 | mg/kg | 2.5 | 2.5 |
| Nicotinic acid | mg/kg | 50.17 | 50.043 |
| Oleic acid C-18: 1 | mg/kg | 10950 | 10950 |
| Palmitic acid C16: 0 | mg/kg | 2700 | 2700 |
| Palmitoleic acid C-16: 1 | mg/kg | 2.5 | 2.5 |
| Pantothenic acid | mg/kg | 50.106 | 50.027 |
| Pentadecanoic C-15: 0 | mg/kg | 2.5 | 2.5 |
| Phenylalanine | mg/kg | 7171.97 | 11759.017 |
| Phosphorus | mg/kg | 7522.765 | 533.069 |
| Polysaccharide | mg/kg | 471700 | 513151.75 |
| Potassium | mg/kg | 7088.682 | 5200.773 |
| Proline | mg/kg | 12762.98 | 9855.784 |
| Selenium | mg/kg | 0.334 | 0.283 |
| Serine | mg/kg | 5267.8 | 10880.31 |
| Sodium | mg/kg | 2488.262 | 2076.593 |
| Stearic acid C-18: 0 | mg/kg | 1250 | 1250 |
| Sulfur | mg/kg | 2791.54 | 3180.027 |
| Threonine | mg/kg | 7154.17 | 8376.773 |
| tricosanoic | mg/kg | 2.5 | 2.5 |
| Tryptophan | mg/kg | 1976.96 | 2127.1 |
| Tyrosine | mg/kg | 9285.01 | 8266.867 |
| Valine | mg/kg | 3296.14 | 10181.275 |
| Vitamin A | I.E./kg | 15000 | 15000 |
| Vitamin B1 | mg/kg | 20.04 | 20.01 |
| Vitamin B12 | mg/kg | 0.03 | 0.03 |
| Vitamin B2 | mg/kg | 20.322 | 20.081 |
| Vitamin B6 | mg/kg | 15.034 | 15.009 |
| Vitamin C | mg/kg | 20 | 19.5 |
| Vitamin D3 | I.E./kg | 500 | 0 |
| Vitamin E | mg/kg | 163.9 | 163.6 |
| Vitamin K3 (as Menadione) | mg/kg | 10 | 5 |
| Zinc | mg/kg | 29.299 | 31.433 |
